# Divergent effects of Wnt/β-catenin signaling modifiers on the preservation of human limbal epithelial progenitors according to culture condition

**DOI:** 10.1038/s41598-017-15454-x

**Published:** 2017-11-10

**Authors:** Hyun Jung Lee, J. Mario Wolosin, So-Hyang Chung

**Affiliations:** 1 Department of Ophthalmology and Visual Science, Catholic Institute of Visual Science, College of Medicine, The Catholic University of Korea, Seoul St. Mary’s Hospital, Seoul, Republic of Korea; 2 Department of Ophthalmology, Eye and Vison Research Institute and Black Family Stem Cell Institute, Icahn School of Medicine at Mount Sinai, New York, NY United States of America

## Abstract

Wnt signaling plays an important role in the regulation of self-renewal in stem cells. Here we investigated the effect of CHIR99021, the primary transducer of the Wnt signaling canonical pathway, and IWP2, a wide action Wnt signal blocker, on the growth and differentiation of the limbal epithelial progenitor cells when these cells are cultured in two different, common culture approaches, outgrowth from limbal biopsy explants and isolated cell seeded in low calcium medium. Consistent with their expected effects, irrespective of the culture system, IWP2 decreased total β-catenin while CHIR99021 increased it in nuclear localization. However, IWP2 increased stem/progenitor cell marker (p63α and ABCG2) content and clonogenic capacity in the explants but had opposite effects on isolated cells. CHIR99021 reduced the growth rate, stem/progenitor cell marker content and clonogenic capacity in the explants but also had the opposite effect on the isolated cells. These results show that the outcome of Wnt/β-catenin signaling modification is dependent on the culture systems. Transplantation of limbal epithelial sheets from explant cultures is one of the standard treatments of limbal stem cell deficiency. Our study shows that Wnt-associated activity has a strong negative impact on stem/progenitor cell preservation in limbal explant cultures.

## Introduction

The corneal epithelium is maintained by limbal stem cells (LSCs)^1^. As with other stem cells, these cells are endowed with the capacity for self-renewal and an extended proliferative potential. However, under normal conditions of corneal epithelial renewal they exhibit slow or infrequent cycling rates. Short-term cell supply is provided by the rapidly proliferating transient amplifying cells (TACs), which derive from LSCs, most likely by asymmetric stem cell division^1,2^. Thus, due to the limited proliferative range of the TACs, the survival of the whole limbal-corneal epithelium is ultimately dependent on its stem cells. Dysfunction of these cells or their outright loss results in limbal stem cell deficiency (LSCD), a condition characterized by impaired corneal wound healing, conjunctivalization of the cornea, and ultimately, partial or total visual loss^3^.

In the prevalent case of unilateral LSCD, regeneration of the damaged ocular surface can be achieved by the autologous transplantation of epithelial sheets generated by expansion of a small amount of limbal epithelial cells from small biopsies of limbal tissue of the healthy contralateral eye using a number of different approaches to achieve expansion. Over the past decade, the generation of these transplantable epithelial cells sheet by cellular outgrowth from biopsy has achieved an appreciable success rate for ocular surface reconstruction and visual outcome^4–6^. Explants have thus become the most common approach for the expansion of limbal epithelial cells for the treatment of LSCD conditions. The high rate of success seems to derive from the fact that the slow cycling status of LSCs is abrogated during cornea epithelial wound healing to provide the extra TACs needed to speed wound closure. There is sound evidence that during such temporary event, whether *in vivo*^7^ or *ex vivo*^8^, LSCs multiply within their niche in such a manner that cells displaying stem/precursor cell features undergo a large expansion within the limbal niche and, subsequently, in the epithelial cells that outgrow from limbal biopsies when those are set in explant culture. Another successful approach includes the growth of epithelial cells enzymatically isolated from the limbal biopsy^9,10^. In this case growth in medium with physiological [Ca^2+^] and serum requires the support of a layer of 3T3 cells. In the absence of feeder cells, epithelial cell growth can be accomplished in serum-free, low [Ca^2+^] media. In these media the calcium-dependent transition to the terminal differentiated is markedly slowed^11^.

Failure of cultivated limbal epithelial sheet transplantation to overcome the LSCD condition may relate to the absence of cells capable of reestablishing a limbal stem cell niche^12^. Consistent with this concept, the success of early limbal epithelial outgrowth sheet transplantation have been shown to correlate with the expression of p63^12^, a protein that plays an essential role in the maintenance of proliferative potential of epithelial cells of ectodermal origin^13^. The p63 expression level seem to correlate with the level of stemness in human^14^ and rabbit^15^ limbal-corneal epithelial lineage. Thus, optimization of culture conditions to increase the percentile of stem/precursor cells within limbal explant outgrowth sheets is critically important to improve the outcome of transplants for LSCD and, given the risks of iatrogenic damage, to minimize the size of the contralateral limbal biopsy used to remediate unilateral LSCD.

The Wnt/β-catenin pathway has been implicated in the regulation of stem cell self-renewal and the fate and embryogenesis of a variety of tissues^16–18^. Under homeostatic conditions, most of β-catenin, a mediator of the main or canonical Wnt signaling pathway, is bound in the cytosol to proteins of the cadherin family^19^. The cytosolic kinase GSK-3β is the principal determinant of β-catenin cellular level. It continuously phosphorylates nascent β-catenin targeting it for degradation. Thus, inhibition of GSK-3β results in increases in β-catenin. Some of the excess protein then becomes available to diffuse into nuclei where it can release the transcriptional activity of TCF4^20–22^. Accordingly, GSK-3β inhibitors are commonly used to induce an increase in β-catenin by mimicking the effects of Wnt in its canonical pathway^23–25^. Newly synthetized Wnt polypeptides undergo palmitoylation in the Golgi apparatus, a derivation that is essential for their activation. Palmitoylation is dependent on the activity of porcupine (porcn), a Golgi membrane O-acyltransferase. Inhibition of this enzyme prevents Wnt release from the Golgi body and thereby abrogates or reduces auto- or paracrine Wnt activity^26^.

Consistent with frequently observed pro-growth and/or anti-differentiation effects of activation of the Wnt pathway on embryonic and somatic stem cell renewal, limbal epithelial cells grown from isolated cells in a low calcium medium, which minimize epithelial cell terminal differentiation while supporting proliferation^11,27^ and, conversely ablation of the β-catenin transcription factor TCF4, reduces clonal cell capacity^28,29^. Additionally, inhibition of GSK-3β with LiCl enhances stem cell features of limbal epithelial cells grown in low calcium medium under support of 3T3 feeder cells^30^. Given the wide utilization of the limbal explant outgrowth cultures to generate limbal epithelial sheets for the treatment of LSCD, we examined the impact of two reagents, the highly specific GSK-3β inhibitor CHIR99021, and IWP2, a highly specific inhibitor of porcn, in this culture. The results were unexpected in that, inhibition of Wnt activity has positive effects in the preservation of stem/precursor cell features in the outgrown cells and conversely, activation of β-catenin led to rapid loss of these features and enhanced differentiation. Therefore we completed a comparative study of the effects of the same reagents on explant outgrowth and isolate cells grown in a low [Ca^2+^] serum free medium. The results show that Wnt/β-catenin signaling modifiers affect the two culture systems in opposite ways. In the limbal niche–supported dense explant outgrowth condition in normal [Ca^2+^], activation of β-catenin and inhibition of porcn have major negative and positive effects, respectively, on outgrowth rate and content of cells displaying stem/precursor features, whereas in low calcium isolated limbal epithelial cell cultures the same actions have the inverse effect. These findings raise intriguing questions on the effect of the cellular context on the processes and effects of Wnt/β-catenin signal transduction. They also have important practical value for the optimization of conditions that inhibition of the Wnt pathways by IWP2 has a positive impact on regeneration capacity of limbal epithelial sheets from limbal explant culture outgrowth in LSCD patients.

## Results

### Effect of Wnt signaling modifiers on cell growth

**Figure 1:**
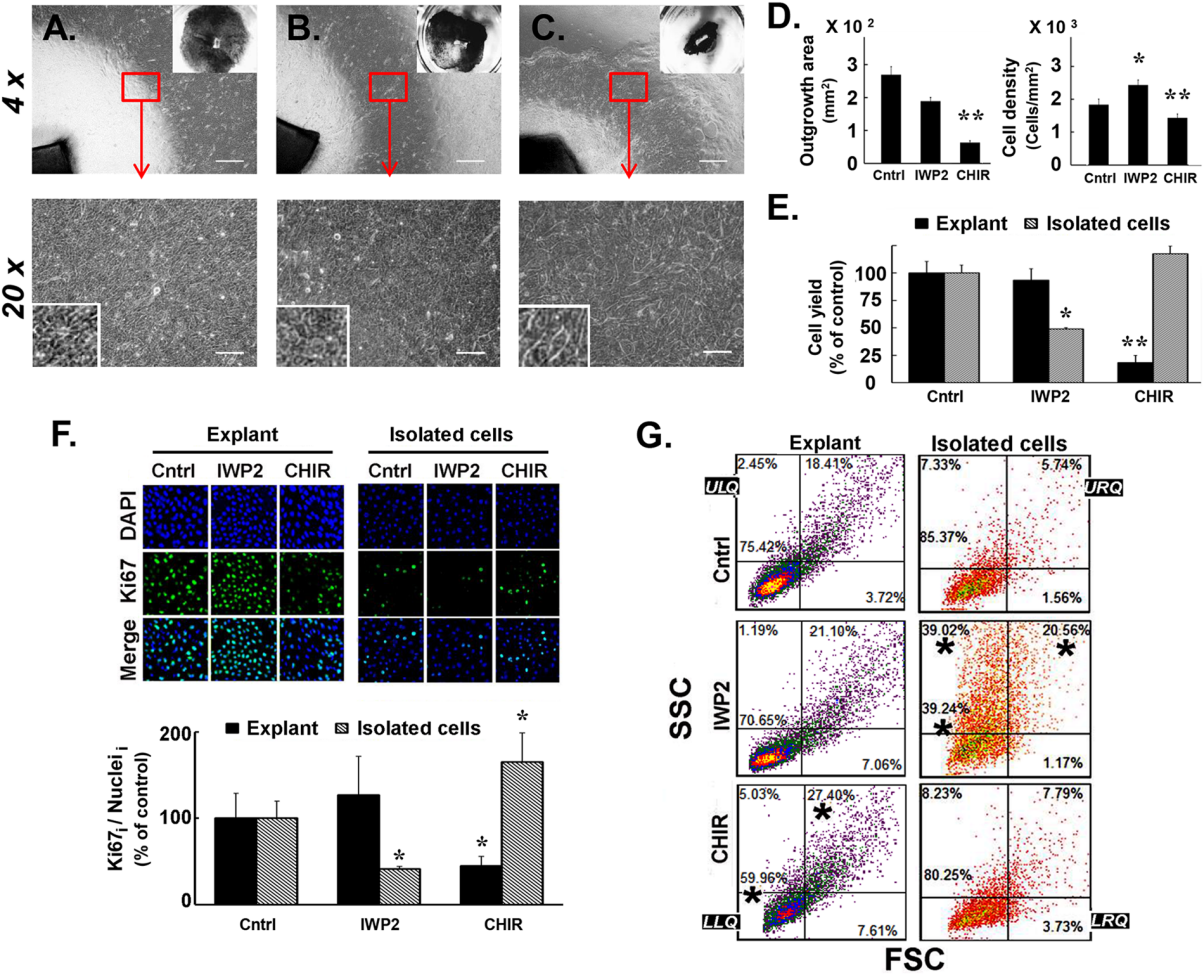
Effect of Wnt signaling modifiers in the growth and proliferative capacity of limbal cell cultures. **(A–C**) Representative micrographs of limbal explant biopsies and their outgrowths in culture medium complemented with DMSO carrier (A, control; Cntrl); 10 μM IWP2 (**B**); or 3 μM CHIR99021 (C; CHIR). Inserts, top right: Photographs of outgrowth cultures stained with Commassie blue R solution. Main frame: phase contrast micrographs obtained with 4x and 20x objectives. Bar = 50 µm. Inserts are 2x larger views of a field in each micrograph. (**D**) Average areas of outgrowth, and cell densities in limbal explant outgrowth after 8 days in control or complemented medium. (**E**) Comparison of relative cell yields in limbal explant outgrowth and isolated cell cultures. (**F**) Immunostain of Ki67 in both culture systems. Representative confocal images and a bar graph of average Ki67 fluorescence intensity stain (Ki67_i_) normalized by the average DAPI intensity for 3 independent cultures are shown. (**G**) Relative size (FCS) and granularity (SSC) of cell recovered from limbal explant outgrowth or isolated cell cultures. Color density flow cytometry plots with different color themes were used for each culture modality. Quadrant (LLQ, ULQ, LRQ and URQ) analysis identified quadrants showing statistically significantly difference (asterisks) with the control values in either the IWP2 or the CHIR99021 complemented cultures. Control, Cntrl; CHIR99021, CHIR, *p < 0.05, **p < 0.01 vs. control (n = 9 from 3 donors).

Limbal explant cultures from biopsy were made as described in Methods. Outgrowths with 10 µM IWP2 were marginally smaller than those in the control medium (Fig. 1A and B) and were similar in appearance under the phase contrast microscope. Outgrowths in the presence of 3 μM CHIR99021 were markedly smaller but individual cells were clearly larger or wider than those grown in the other two conditions (Fig. 1C). Figure 1D shows the mean size ± SD of the outgrowth area (left) and the cell density (right; cell yield/growth area) for all three conditions in the explant cultures. With regard to the cell yield, in the explant cultures it was significantly lower in the CHIR99021-treated cultures (p < 0.01), but there was no statistically significant difference in cell yields between control and IWP2-treated cultures; the smaller growth area for this latter condition reflects the higher cell density of the explant cultures in the presence of IWP2 (Fig. 1E). Conversely, cell density was significantly lower in the CHIR99021-treated outgrowth (Fig. 1E, p < 0.01).

Two observations indicate that proliferation differences are the primary driver for the differences in the cell population growth. The first is the differences in the content of the proliferation marker Ki67 between the different samples showing differences in growth rate (Fig. 1F). In the explant cultures the inhibitory effect of CHIR99021 was concurrent with a large (>60%) decrease in Ki67 staining; in the isolated cell cultures, in turn, the growth inhibitory effect of IWP2 coincided with a similarly large decrease of Ki67 stain. The second supporting fact is that in all these cultures floating cells never amounted to more than 2% of total amount of cells harvested by trypsinization, thus cell loss is unlikely to be a substantial factor for cell yields.

Finally, the higher cell density induced by IWP2 in explant culture system may result from either higher cellular compaction (i.e., cells adopting a more columnar shape), or smaller cells, or both. To discriminate between these two possibilities we examined the light scattering properties of the outgrown cells by flow cytometry (Fig. 1G). In bivariate analysis, we assessed FSC (proportional to cell size) and SSC (proportional to granularity or intracellular complexity) using arbitrarily selected quadrants (LLQ) to provide a semi-quantitative view of changes induced by the Wnt signaling modifiers. The numbers in each quadrant represents the results for the experiment displayed and asterisks identify quadrants where the experimental sample was statistically different (p < 0.05) from its respective controls in either culture modality. In the explant cultures there was no statistical difference in the quadrant distribution of control and IWP2-treated cells, though IWP2 decreased the average FSC by 16.7 ± 1.4% (p < 0.05). In contrast, CHIR99021 exposure increased simultaneously both cell size and granularity. In the isolated cell cultures, the effects were diametrically different. CHIR99021 was not significantly different from its control in either quadrant distribution or average FSC and SSC whereas IWP2 markedly increased cell granularity (SSC).

### Effect of Wnt signaling modifiers on β-catenin and TCF4

As described in the Introduction, earlier studies on the effect of the Wnt signaling modifiers in isolated cell cultures, whether grown in serum free low calcium medium or under 3T3 feeder cells support, have shown pro-growth and anti-differentiation effects of Wnt signaling activation. Given that the effects of IWP2 and CHIR99021 in limbal explant outgrowth cultures indicated an inverse Wnt-growth relationship, we examined whether the difference in the two culture systems might be related to opposite effects of Wnt signaling modifiers on β-catenin, the principal mediator of the canonical Wnt signaling pathway.

**Figure 2:**
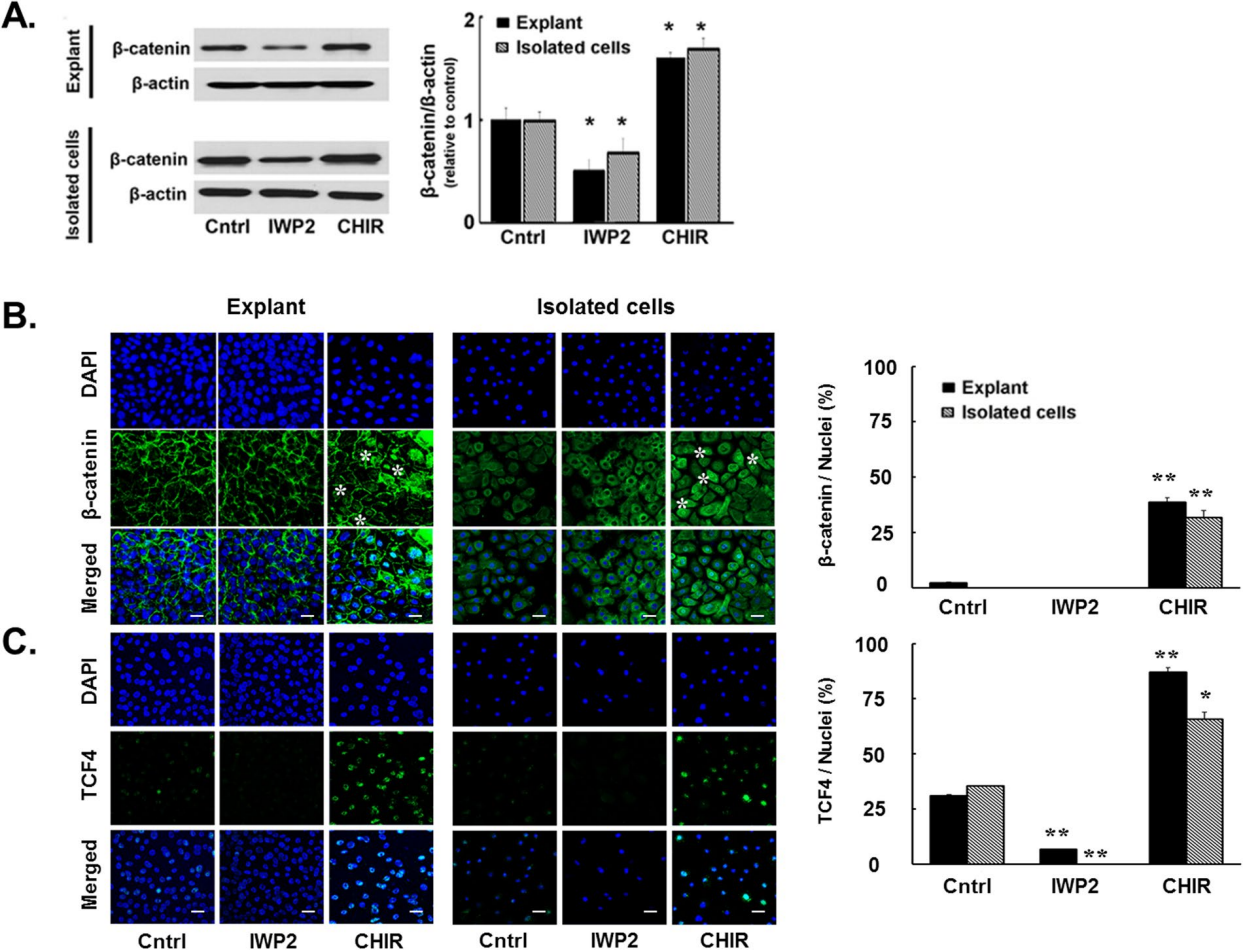
Effect of Wnt signaling modifiers on β-catenin and TCF4. (**A**) Representative Western blots and side-by-side bar graph of mean ± SD of total β-catenin protein. The protein expressions of total β-catenin and β-actin were developed from the same gel in western blot. (**B**) Representative immunostained images and statistical data for β-catenin. Addition of CHIR99021 causes the appearance of an annular nuclear stain in the great majority of cells in both culture systems (selected cells indicated by asterisks). (**C**) Representative immunostained images and statistical data for TCF4. In both culture systems, control cells show weak nuclear staining and addition of CHIR99021 increases the percent of cells with overt TCF4 staining. Control, Cntrl; CHIR99021, CHIR, *p < 0.05, **p < 0.01 vs. control (n = 6 from 2 donors).

We compared the effect of IWP2 and CHIR99021 on total β-catenin levels in either culture system. In both culture systems IWP2 caused a statistically significant decrease of total β-catenin level whereas CHIR99021 caused a statistically significant increase of levels (Fig. 2A). Immunostaining of explant outgrowth cultures showed that the overwhelming majority of the β-catenin protein in the control condition is localized almost exclusively at the plasma membrane (Fig. 2B), in which it is known to be associated with cadherin^31^. IWP2 did not cause a distinguishable change in this pattern but under CHIR99021, most cells acquired nuclear stain the β-catenin. This latter result is consistent with the notion that inhibition of GSK-3β by CHIR99021 prevents phosphorylation of β-catenin and its degradation leading to an increase in the total amount of β-catenin and accumulation of the stable, de-phosphorylated form in the nuclei. In the low calcium-isolated cell cultures, in which cell adhesions and cadherin function are inhibited^32^, β-catenin was diffusely present throughout the cytosol. This staining pattern was not modified by IWP2, whereas CHIR99021 induced the same nuclear accumulation of β-catenin observed in the explant cultures. In the isolated cell cultures, the stain in control and IWP2 treated conditions is primarily cytosolic. In the nuclei, β-catenin can recruit the transcription factor TCF4 to affect the cell transcription pattern^20–22^. Consistent with this notion, staining for TCF4 (Fig. 2C) revealed that CHIR99021 increased the extent of TCF4 nuclear stain in both culture systems by 2–3 folds relative to the control. These results provide strong indication that the GSK3/β-catenin/TCF4 interactions are very similar in the two culture systems. Thus, the divergence of cell proliferation effects by IWP2 and CHIR99021 between the two cultures documented in Fig. 1 do not appear to be related to the divergence in the primary elements of the canonical Wnt signal transduction chain.

### Effect of Wnt signaling modifiers on stem/progenitor cell markers and clonogenicity

**Figure 3:**
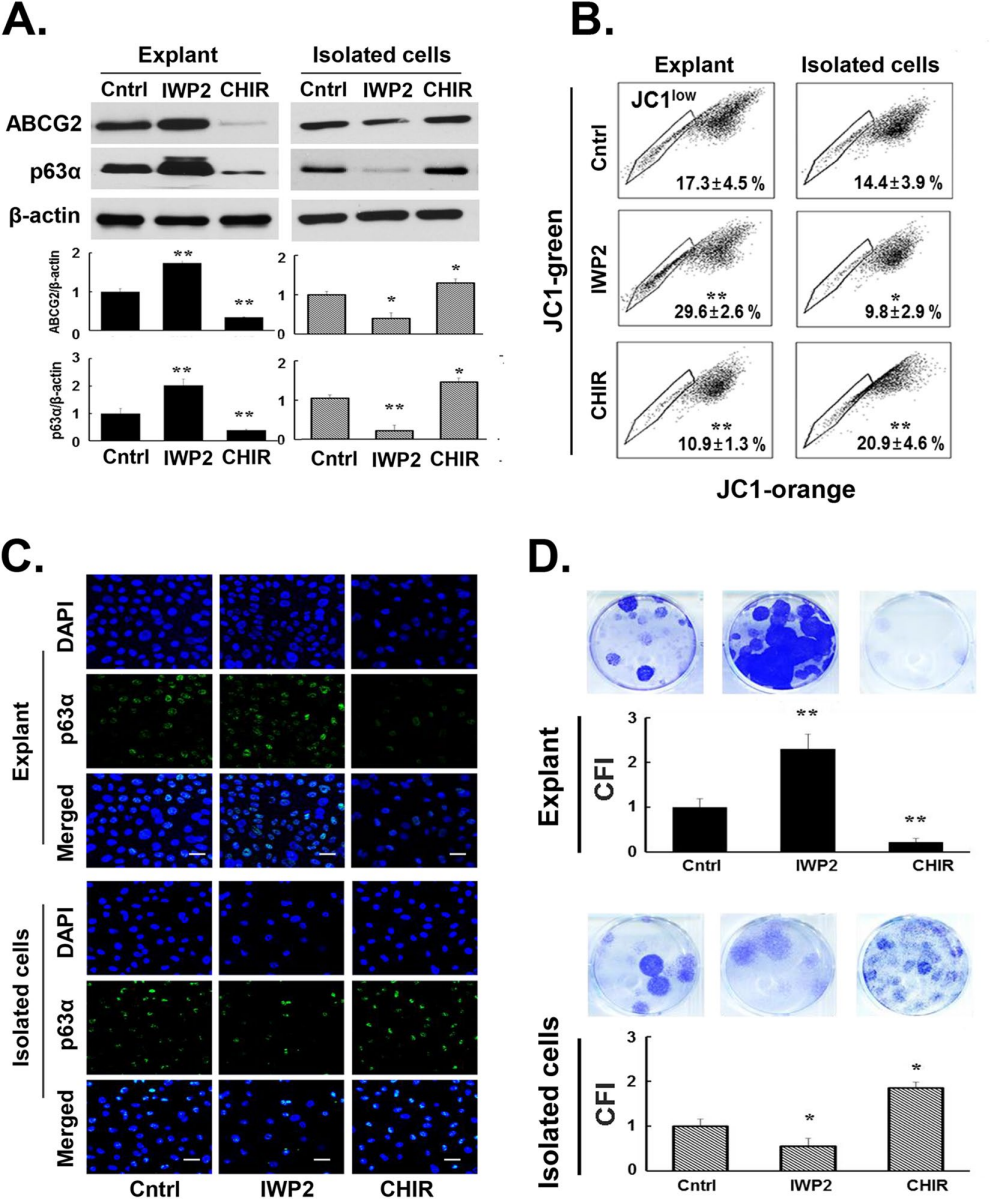
Effect of Wnt signaling modifiers on stem/progenitor cell marker expression and population clonal index. **(A**) Top. Representative Western blots of ABCG2 and p63α. Bottom. Statistical result for both markers. All signal intensities were normalized to the signal generated in the same sample by β-actin. The protein expressions of ABCG2, p63α and β-actin were developed from the same gel in western blot. Note that both IWP2 and CHIR99021 have strong effects on stem/progenitor cell markers contents but the effects are in the opposite direction on the two distinct culture systems. (**B**) JC1 green/orange bivariate emission plots. Dye exclusion reflects ABCG2 activity and result in the JC1^low^ cohort. Mean ± SD for three independent experiments are shown within the plots. (**C**) Representative images for p63α immunostaining. All staining is in nuclei. These images point to a strong decrease in antigen content by CHIR99021 in the explant outgrowth cultures and by IWP2 in the isolated cell cultures. (**D**) Representative images and mean ± SD for colony formation indices (CFI), normalized to the control CFI for cells recovered from explant outgrowths or isolated cell cultures. Note the opposite effects of the two Wnt signaling modifiers with respect to each other and with respect to each culture modality. Control, Cntrl; CHIR99021, CHIR, *p < 0.05, **p < 0.01 vs. control (n = 9 from 3 donors).

Prior studies of Wnt signaling effects on isolated epithelial cell culture modalities demonstrated that GSK-3β inhibitors increase the expression of stem/progenitor cell markers^29,30^. To determine whether the growth effects of IWP2 and CHIR99021 had their counterparts in the cell phenotype, we examined the expression of stem/progenitor cell markers, the xenobiotic efflux transporter ABCG2 and p63α, which is associated with limbal epithelial cells stemness and proliferation potential (Fig. 3A). Western blots showed that in the explant cultures IWP2 caused statistically significant 70–80% increases in ABCG2 and p63α protein content whereas CHIR99021 reduces the two proteins by similarly large percentiles. In contrast, in the isolated cell cultures IWP2 caused a statistically significant decrease of these two proteins and CHIR99021 caused a small amount of increase which is statistically significant.

We also analyzed the actual ABCG2 activity (Fig. 3B). In the explant cultures IWP2 induced a 71.1% (p < 0.01) increase in the percent of cells resilient to full staining by the ABCG2 substratum JC1 (JC1^low^) and, fittingly, the strong reduction of ABCG2 protein generated by CHIR99021 was matched by a 37.0% decrease in the percent of cells resilient to this staining compared with control (p < 0.01). Coinciding with the effects of the two Wnt signaling modifiers on ABCG2 protein content when applied to the isolated cell cultures, IWP2 and CHIR99021, respectively, decreased (i.e., smaller JC1^low^ fraction) and increased ((i.e., larger JC1^low^ fraction) efflux transport function.

Immunostaining for p63α produced images that were fully consistent with the Western blot study (Fig. 3C). Namely, in the explant cultures IWP2 and CHIR99021, respectively, increased or decreased the number of nuclei stained for this antigen and/or the average intensity of the stain but caused the opposite effects on the isolated cell cultures.

Finally, we compared the effect of IWP2 and CHIR99021 on colony formation indices (CFI) (Fig. 3D). For these experiments, the harvested cells from explant cultures and isolated cell cultures were then cultured in low calcium medium side-by-side under clonogenic, low-density conditions as described in Methods. As with the phenotypic parameters, IWP2 and CHIR99021 had divergent effects on the preservation or expansion of colonies. IWP2 increased the clonogenic capacity of explant outgrowth cell populations while CHIR99021 decreased their clonogenic capacity. As with the other markers described above the activating and inhibiting roles of these two reagents were reversed in the isolated cell cultures.

### Effect of Wnt signaling modifiers on Krt12 expression

**Figure 4:**
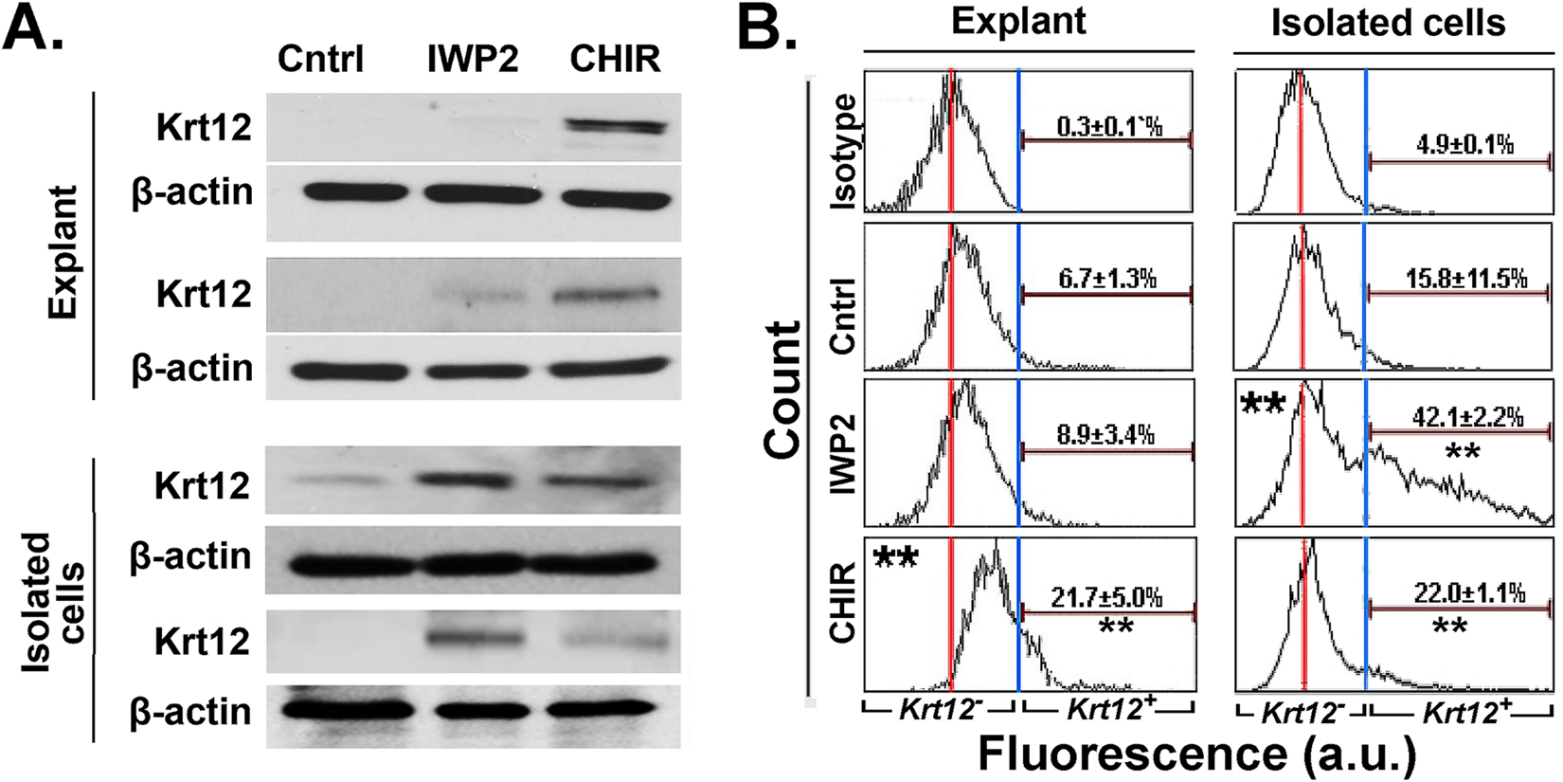
Effect of Wnt signaling modifiers on corneal epithelial differentiation marker Krt12 content. (**A**) Duplicate (independent samples) Western blots for Krt12 and β-actin from explant outgrowth cultures and isolated cell cultures. The protein expressions for each independent sample of Krt12 and β-actin were developed from the same gel in western blot. Note that the explant outgrowth cultures in control medium do not express measurable amounts of Krt12 and that CHIR99021 causes increases in Krt12 in both culture systems. (**B**) Intracellular flow cytometry of cells harvested and stained for Krt12. The fluorescence scale is logarithmic. Arbitrary thresholds used to define Krt12^−^ and Krt12^+^ cells are represented by vertical blue lines. The vertical red lines indicate the maximum for the intrinsic fluorescence frequency. Mean ± SD of Krt12^+^ cells for 3 independent experiments are given inside the histograms. Note that a) in the explant outgrowth cultures CHIR99021 increase both the percent of cells within the zone defined as Krt12^+^ and the intensity of the population defined as Krt12^−^ and b) in the isolated cell cultures both IWP2 and to a lesser extent the CHIR99021 increase Krt12^+^ cells. Control, Cntrl; CHIR99021, CHIR, **p < 0.01 vs. control (n = 9 from 3 donors).

Western blot (Fig. 4A) and intracellular flow cytometry (Fig. 4B) were concomitantly used to measure the expression of the corneal differentiation marker keratin 12 (Krt12). In control condition in both culture systems, Krt12 was either undectable or minimally present. Intriguingly, unlike the opposite effects for CHIR99021 and IWP2 documented in Fig. 3 for the multiple stem/progenitor markers whether in the explant or the isolated cell cultures, both reagents caused increases in Krt12, albeit to different degrees. In the explant outgrowth cultures, CHIR99021 caused strong overt expression of Krt12. Because of the near absence of Krt12 expression in the control samples, it was not possible to make determinations of fold change for this difference. The qPCR demonstrated a matching 2.5 fold-increase of Krt12 mRNA levels (data not shown). The equivalent results for IWP2 indicated the possibility of slight increases in Krt12 expression in the Western blots of (Fig. 4A second specimen; compare Control vs. IWP2) matched by a slight increase in the percent of cells exceeding the arbitrary intensity threshold used in flow cytometry (Fig. 4B; compare Control vs. IWP2). However, we were not able to establish a statistically significant difference with the control. In the isolated cell cultures, IWP2 caused a very large increase in total Krt12 expression by Western blot (Fig. 4A) underpinned by a 3–4 fold increase in the number of cells within the Krt12^+^ domain (Fig. 4B). Notable, in spite of its pro-growth effects in the isolated cultures, CHIR99021 also caused some increase in Krt12 expression driven by a small, but statistically significant increase in the percent of cells within the Krt12^+^ range.

## Discussion

The presence of limbal stem/progenitor cells within limbal epithelial sheets generated by explant culture is critical for the success of cultivated limbal epithelial sheet transplantation for the treatment of LSCD. Thus, manipulation of Wnt signaling represents an attractive resource for the improvement of limbal stem/progenitor cell content or phenotype in these cultures. Based on the facts that a) in isolated limbal epithelial cell cultures in low calcium medium TCF4- or LiCl help preserve the stem cell phenotype or inhibits differentiation^28–30^ and b) that multiple other studies has linked activation of the Wnt signaling pathway with the survival or self-renewal of multiple somatic and embryonic stem cell–based organs^32–34^, we anticipated that our study in the limbal explant outgrowth cultures from limbal explants would also show a positive correlation between increased β-catenin levels and stem/progenitor cell enrichment.

Instead, increased β-catenin in the explant culture conditions resulted in unexpected outcomes, outgrowth rate and cell yield were reduced, the cell size grew, and the expression of p63α, ABCG2 and clonogenicity decreased. In addition, increased β-catenin levels and its nuclear translocation led to a large increase in Krt12-positive cells. The seemingly unexpected outcome, though, is not unique; negative correlations between activation of Wnt-signaling and growth function had been reported earlier, including in the differentiation of embryonic stem cells^35^ and, most significant, in epidermal wound healing^36^. A process of epidermal wound healing shares with the limbal explant outgrowth multiple spatial and biological features including cell migration from the edge of the epithelial-free area, continuous in very short range cell-cell interactions via adherent and gap junctions, mechanical forces transmitted through the cytoskeleton and restricted intercellular spaces that would enhance paracrine function of locally secreted cytokines or growth factors. The observation that IWP2, which is expected to inhibit most endo-, paracrine Wnt activity, enhanced stem/progenitor cell features and clonogenic cell content is consistent with the notion of a negative impact of the canonical Wnt activity and survival of stem/progenitor phenotype in the explant culture. However, the possibility that the relatively subtle IWEP2 effects may wholly or partially reflect a concurrent inhibition of non-canonical Wnt activities or even unrecognized effects of porcn on other cell functions cannot be presently discounted. The complexities of Wnt signal transduction that lead to opposite outcomes in different experimental cells or systems has been recently critically discussed by Lien and Fuchs^18^.

In the isolated cells cultured in low calcium, both the pro-growth effect of CHIR99021 as well as the strong growth-inhibitory and differentiation-inducing effect of IWP2 support the conclusion of the previous studies on isolated limbal epithelial cells, namely that β-catenin and TFC4 play an essentially positive role in the survival and expansion of the stem/precursor phenotype in such culture conditions^30^.

The divergent outcomes of Wnt/β-catenin manipulations in the different culture systems for *ex vivo* limbal epithelial stem/progenitor cell expansion may reflect the considerable differences in the cellular context for growth initiation and population expansion. The fundamental event for the culture of isolated limbal epithelial cells in low calcium is likely to be the initial proliferation event. This cell division occurs in relative isolation from other epithelial cells in particular at low densities; β-catening-TCF4 signaling in these conditions may be critical for the survival of the isolated cells^24^. In contrast, initial expansion in explant cultures is likely to be determined by the migratory ability of cells from explant to growth substratum. The phenotype of the outgrowing cells depends on the relative mobility of cells that populate the basal layer of the limbal epithelium as they migrate into the substratum. We have previously shown that the early outgrowth is populated by more differentiated cells^6^. This cellular distribution makes sense because both rapid proliferative and migratory responses to the wound-like condition of the explant are expected to occur in the short-term repopulating TA cells. If Wnt activation further enhances TAC advantage in the rapidity of the initial response, these cells, which are likely to undergo terminal differentiation in short sequence, will crowd out the outgrowth of the more valuable progenitors.

An alternative explanation for the effect of CHIR99021 on the explant cultures may involve unintended consequences of GSK3-3β inhibition or secondary effects of the activation of the β-catenin/TCF transcriptional complex. In this context the putative increased activity of the c-myc oncogene should be particularly considered. Increases in c-myc may occur in our cultures in two distinct manners. Firstly, GSK-3β phosphorylation of ERK-activated S62-phosphorylated c-myc is required for its conversion to the unstable T58-phosphorylated form^37,38^, hence GSK-3β inhibition can be expected to reduce c-myc degradation rate. C-myc is a main transcriptional target of the β-catenin/TCF complex, so that as the complex activity increases so may c-myc activity^39^. In turn, myc simultaneously activates epidermal stem cells proliferation and accelerates the rate of differentiation of the stem cell progeny^40^. Thus, the divergent outcomes of Wnt signaling manipulation may reflect differential impact of c-myc or other unrecognized signal transduction components in the two culture systems. Recently, increased c-myc activity has been invoked as the reason for which Wnt activation induces differentiation within the hair follicle^41^. Finally, the involvement of c-myc hypothesis could explain the observation that while CHIR99021 causes inverse effects on the two culture systems in multiple stem/progenitor cell-associate parameters, it increases Krt12 expression in both systems: increased myc activity following inhibition of GSK-3β may foster the activation of stem/precursor cells and may concurrently induce accelerate differentiation of late TAC cells present in the cell population.

Considering the widespread clinical application of explant outgrowth cultures for the treatment in limbal stem cell deficiency^6,7,42^, understanding the cell-molecular sources of the divergent effects of Wnt signaling modifiers may help develop better regenerative biological strategies or pharmacological treatments for intractable ocular surface diseases.

## Methods

### Tissue procurement

Post-keratoplasty discards of human corneal-limbal tissues from unidentifiable cadavers were obtained from Seoul St. Mary’s Hospital Eye Bank (Seoul, Korea). The Institutional Review Board determined that use of these tissues did not constitute research on human subjects. Tissue acceptance criteria included (1) donor age of 30–65 years at death; (2) tissue harvest occurred within 12 h of death; (3) tissue was stored in Optisol less than 48 h after harvest; and (4) the donor tested negative for human immunodeficiency virus, hepatitis B or C, Epstein-Barr virus, and syphilis. With the use of a dissecting microscope placed on a horizontal sterile air flow hood, corneas were initially split into eight equal segments, then the conjunctival edge of each segment was dipped in 1% trypan blue (Sigma-Aldrich, St. Louis, MO) to stain the conjunctival stroma, and all remnants of conjunctiva were carefully removed using conjunctival scissors. After scleral tissue was trimmed away, 0.5-mm-wide limbal strips were cut away from each corneal segment with a sharp blade.

### Culture of limbal biopsy explant

Two mm long human limbal segments were set up epithelial -side up on the center of 0.4-μm-pore 25-mm-diameter polyester membrane inserts (Corning Costar, Corning, NY). The inserts were pre-equilibrated in a supplemented hormonal epithelial medium (SHEM) consisting of 950 ml of a 1:1 mix of Dulbecco’s modified minimal essential medium and Ham F12 (DMEM/F-12, Gibco, Grand Island, NY), 50 ml of fetal bovine serum (FBS, Gibco), 5 ng of human recombinant epidermal growth factor, 28 mg of phosphoethanolamine, 1x ITS (Gibco) and 1x penicillin-streptomycin (Gibco) mixes alone (control medium) or complemented with 10 µM IWP2 (Tocris, Bristol, UK) or 3 µM CHIR99021 (Stemgent, San Diego, CA). A single batch of FBS was used in all experiments. Culture media were refreshed every 72 h. When outgrowths in control medium reached 70–80% confluence (typically in 10–14 days), outgrown cells were incubated in trypsin (Gibco) for 10 min to obtain fully dissociated cells. Cells were pelleted, resuspended in SHEM, and counted in a hemocytometer. Some cultures were washed with PBS, fixed-stained in 10% acetic acid–45% methanol containing 0.45% Commassie blue R 250, and photographed. Whenever a source is not indicated for a chemical, it was purchased from Sigma.

### Culture of isolated limbal epithelial cells in low calcium medium

Limbal biopsy segments were incubated at 4 °C overnight with 2 mg/ml of Dispase II (Roche) in KSFM (Invitrogen, Carlsbad, CA) supplemented with 10% FBS. The limbal epithelium layer was then gently sloughed off from its stromal base under a dissecting microscope, incubated in TrypLE (Sigma) for 10 min and triturated with a pipette to generate an isolated cell suspension. These cells were seeded at a rate of 10^4^ cells/cm^2^ in Cnt-Pr (Cell-N-Tec, Bern, Switzerland) complemented with bovine pituitary extract on 25 cm^2^ in T-flask coated with bovine collagen solution (PureColl^tm^, Biomatrix, San Diego, CA).

### Flow cytometry

Cells were resuspended in FACS buffer consisting of 95% 4-(2-hydroxyethyl)-1-piperazineethanesulfonic acid (Hepes)–buffered DMEM/F12–5% bovine serum albumin (BSA) and 1 μg/ml propidium iodide (PI) and analyzed by flow cytometry to obtain forward (FCS; proportional to cell size) and side (SSC; proportional to cell granularity/intracellular complexity) light scatter distributions. In addition, we determined ABCG2-dependent efflux activity, a property tightly linked to stemness in multiple somatic cell systems including the limbal-corneal system^43–46^. Explant outgrowth cells and isolated cultured epithelial cells were seeded overnight in SHEM in 6-well plates, incubated for 45 min with the 250 nM JC1 (Axxora, San Diego, CA), released by a 2- to 3-min trypsinization and diluted in FACS buffer (5% FBS in PBS). JC1, a mitochondrial binding dye displaying an accumulation-dependent bathochromic emission shift is an ABCG2 substratum. In cells displaying high ABCG2, its efflux activity prevents JC1 from reaching its mitochondrial binding sites. Thus, in flow cytometry bivariate 531(green)/585(orange) emission plots, these cells appear as a low-stain cohort (JC1^low^) lying on the left of the cohort of fully stained low/nil ABCG2 activity cells^5^.

To stain for Krt12, cells were, a) enzymatically harvested; b) fixed with 10% formalin for 10 min; c) permeabilized with 0.1% Triton X-100 in PBS for 30 min; d) incubated with 5% BSA-PBS for 30 min; d) incubated with a goat polyclonal antibody recognizing Krt12 (Santa Cruz Biotechnology, Santa Cruz, CA) for 45 min; e) incubated, with Alexa-488 conjugated rabbit anti-goat IgG (Thermo Fischer, Waltham, MA) for 30 min; and f) suspended in FACS buffer. Between each step, cells were diluted in PBS and pelleted down to remove the previous solution. Flow cytometry was performed in a FACS Canto II (BD Biosciences, San Diego, CA) instrument. Results were analyzed by FACS Express (DeNovo, Glendale, CA).

### Western blot

Cells were washed with PBS and then lysed with lysis buffer containing phosphatase inhibitor cocktail 2 (Sigma-Aldrich), and protease inhibitor cocktail (Roche Diagnostics, Indianapolis, IN). Proteins were mixed in sample-loading buffer, boiled for 10 min, and centrifuged before the protein concentration in the clarified lysates was determined using the BCA Protein Assay kit (Thermo Fisher). Equal amounts of protein in cell lysates were separated by 10% SDS-polyacrylamide gel electrophoresis (SDS-PAGE) under reducing conditions and electro transferred to a PVDF membrane (Millipore, Billerica, MA). The membrane was blocked with 5% skim milk in PBS containing 0.1% Tween 20, incubated at 4 °C for 18 h with primary mouse monoclonal antibodies (Abs) recognizing, β-catenin (BD Biosciences), ABCG2 (Abcam, Cambridge, MA), the goat polyclonal Ab targeting the corneal differentiation marker Krt12, or rabbit polyclonal Ab targeting p63α (Abcam), the p63 isoform associated with the limbal epithelial stem cells^46^. After three washes, the membranes were incubated at room temperature for 1 h with the appropriate anti-mouse, anti-goat or anti-rabbit horseradish peroxidase–conjugated secondary Abs (Thermo Fisher). Finally, the membranes were washed three times and protein bands were detected using enhanced chemiluminescence reagent (ECL; Amersham Biosciences, Buckinghamshire UK). All membranes were stripped and reprobed with mouse monoclonal anti-β-actin Ab to provide a normalizing reference.

### Clonal assays

Cells harvested from the limbal explants outgrowths or the isolated cell cultures by trypsinization were seeded in triplicate on bovine collagen type I–coated 6-well plates in Cnt-Pr at a density of 100 cells/cm^2^. The cultures were maintained up to 14 days with medium replacement on days 4, 7, 10, and 13. They were then fixed in cold methanol and stained with the Commassie blue R 250 solution.

### Immunofluorescence

Insert membranes with outgrowth epithelial sheets were separated from their plastic mounts and cells were fixed with cold methanol for 10 min, permeabilized with 0.1% Triton X-100 in PBS for 30 min, and incubated with 10% goat serum for 1 h to block nonspecific reactions. Sections of the outgrowths and cells were then incubated with anti-Ki67, anti-β-catenin, anti-TCF4 (mouse monoclonal, Santa Cruz) or anti-p63α Abs, washed with TBS twice, and incubated with Alexa Fluor 488–conjugated anti-rabbit and mouse IgG Ab (Note: modify if TCF4 is a polyclonal). Stains were captured by confocal microscopy in an LSM 510 Meta confocal microscope (Carl Zeiss, Oberkochen, Germany). The Ki67 and TCF4 stain were quantitated in Photoshop software (Adobe Systems, Santa Clara, CA). The luminosity (i.e., stain intensity) of the background areas (range, 0–255; 8 bit luminosity scale) was determined with the Photoshop histogram function. We counted the number of pixels with luminosity equal to or above the luminosity of the highest 1% of the background range pixels. Blue (DAPI^+^) luminosity is assumed to be proportional to the number of nuclei present in the field and hence a relative measure of nuclear count. Alexa 488 luminosity is assumed to be proportional to the number of antigen units present in these nuclei and hence the Alexa 488/DAPI ratio is proportional to the density of Ki67 epitopes per nuclei. Three randomly selected areas of equal size and sufficiently large to incorporate at least 100 nuclei were counted to obtain mean ± SD for each condition tested. For graphic representation, all values were normalized by the control value, which was set as equal to one.

### RNA isolation and quantitative PCR

Total RNA was isolated using TRIzol^tm^ reagent (Gibco-Invitrogen). The first strand of complementary DNA (cDNA) was synthesized with random hexamers using SuperScript III^TM^ reverse transcriptase (Invitrogen). Real time PCR was done using CCAGGTGAGGTCAGCGTAGAA and CCTCCAGGTTGCTGATGAGC as forward and reverse primers for human Krt 12 and SYBR Green reagent. Relative quantification was made according to the 2^−ΔΔCt^ method using the GAPDH cycle thresholds (CTs) for each sample for CT normalization.

### Statistics

Statistical significance between groups was examined by a nonparametric, two-tailed Mann–Whitney t-test using SPSS 17.0 version. We regarded p < 0.05 as significant and p < 0.01 as highly significant. Bar graph and flow cytometry mean ± SD values were derived from the values obtained in 3 independent experiments from different donors. The value for each experiment, in turn, was the average of triplicate or, in some cases, duplicate measurements (n = 9 from 3 donors) or (n = 6 from 3 donors).

### Data availability statement

All data from the current study that were generated or analyzed are available upon reasonable request from the corresponding author.
