# Author Correction: Divergent effects of Wnt/β-catenin signaling modifiers on the preservation of human limbal epithelial progenitors according to culture condition

**DOI:** 10.1038/s41598-018-21906-9

**Published:** 2018-03-01

**Authors:** Hyun Jung Lee, J. Mario Wolosin, So-Hyang Chung

**Affiliations:** 1 Department of Ophthalmology and Visual Science, Catholic Institute of Visual Science, College of Medicine, The Catholic University of Korea, Seoul St. Mary’s Hospital, Seoul, Republic of Korea; 2 Department of Ophthalmology, Eye and Vison Research Institute and Black Family Stem Cell Institute, Icahn School of Medicine at Mount Sinai, New York, NY United States of America

Correction to: *Scientific Reports* 10.1038/s41598-017-15454-x, published online 10 November 2017

The Acknowledgements section in this Article is incomplete.

“This work was supported by a grant from the Korea Health Technology R&D Project, Ministry of Health & Welfare, Republic of Korea, HI14C1607 (S-HC), an NIH/NEI RO1-EY014878 and an unrestricted grant from RPB, Inc. (JMW).”

should read:

“This work was supported by a grant from the Korea Health Technology R&D Project, Ministry of Health & Welfare, Republic of Korea, HI14C1607 and by the National Research Foundation of Korea(NRF) grant funded by the Korea government(MSIP) (No. 2017R1A2B4012327) (S-HC), an NIH/NEI RO1-EY014878 and an unrestricted grant from RPB, Inc. (JMW).”

